# School Matters: The Effects of School Experiences on Youth’s Attitudes toward Immigrants

**DOI:** 10.1007/s10964-021-01497-x

**Published:** 2021-09-24

**Authors:** Katharina Eckstein, Marta Miklikowska, Peter Noack

**Affiliations:** 1 Department of Educational Psychology, Friedrich Schiller University of Jena, Humboldtstr. 27, 07743 Jena, Germany; 2 Department of Sociology, Beteendevetarhuset, Umeå University, Mediagränd 14, 901 87 Umeå, Sweden

**Keywords:** Prejudice, Attitudes toward immigrants, School, Classroom climate, Youth

## Abstract

Although schools have been described as an important socialization context for the development of intergroup attitudes, longitudinal multilevel studies are still rare within this field. This 3-wave study (with annual assessments) of German adolescents (*N* = 1292; *M*_age_ = 13.86; 51.8% female) examined the role of school experiences (perceived multicultural education, supportive peer relations in class, democratic classroom climate) in the development of youth’s negative attitudes toward immigrants. Longitudinal multilevel analyses revealed that a democratic classroom climate predicted youth’s attitudes at the individual level. At the classroom level class-average perceptions of a democratic classroom climate, supportive peer relations in class, and multicultural education (the latter solely among male, higher track students) were associated with less negative attitudes toward immigrants. In addition, age moderated the effect of school experiences on attitudes, showing that perceptions of a democratic climate at the classroom level mattered in particular among older adolescents. The findings suggest that school experiences are related to youth’s negative attitudes toward immigrants and can therefore help to reduce the risk of prejudice development.

## Introduction

As societies become increasingly culturally diverse, reports of intolerant attitudes toward immigrants are viewed with concern—a concern that becomes all the more serious when it affects young people. Youth is considered a formative period in life for the development of social and political attitudes (Neundorf & Smets, 2017). Accordingly, besides becoming more aware of one’s own social identities, intergroup attitudes, which reflect relatively enduring and general evaluations of various social groups (APA, n.d.), stabilize (Crocetti et al., 2021). With an increasing political awareness, yet still searching for a sense of identity, young people are particularly susceptible to contextual influences (impressionable years hypothesis; e.g., Sears & Levy, 2003).

Of the many factors that shape young people’s intergroup attitudes, experiences in school deserve particular attention as young people spend much time in educational settings and schools share the common goal of educating students to become informed citizens (Neundorf & Smets, 2017) and to counteract prejudice (Hess, 2009). Moreover, socialization and social learning perspectives (e.g., Bandura, 1977) see schools as miniature societies that bring together young people with various backgrounds and therefore allow learning about social interaction and group processes (Dessel, 2010). Accordingly, studies have shown that school experiences are linked to youth’s intergroup attitudes (Barber et al., 2013). However, the school context offers a variety of influences. Apart from structural characteristics (e.g., cultural diversity), formal learning experiences (e.g., curricular initiatives in multicultural education) can be distinguished from informal learning experiences (e.g., prevailing school or classroom climate; Scheerens, 2011). Since these experiences can be furthermore located at different levels of the school context, ranging from an individual student in a particular classroom or proximate dynamics within class to more distal processes at the school level, an ecological view of school has been proposed (Eccles & Roeser, 2009). Yet, longitudinal studies accounting for the hierarchical nature of the school context are still rare, as are considerations of age-specific trends that would allow examining whether young people are particularly responsive to school influences at a certain age and thus at a certain stage of development. Drawing on longitudinal multilevel data, it was therefore the goal of the present study to examine the effects of formal-curricular and climatic school experiences on German youth’s negative attitudes toward immigrants, while also accounting for potential age-related patterns.

### Curricular School Experiences and Youth’s Intergroup Attitudes

Curricular characteristics reflect an important formal aspect of the school context and one approach that has attracted particular research attention in this regard is multicultural education (Banks & Banks, 2004). The concept subsumes a variety of educational practices ranging from temporary school-based interventions to general approaches to teaching that can be implemented with or without intergroup contact (Aboud & Levy, 2000). More precisely, multicultural education “aims to provide students with knowledge and attitudes necessary to understand, respect, and interact harmoniously as equals with members of different ethnic groups” (Aboud & Levy, 2000, p. 277). It can promote norms of tolerance, thereby helping young people to look beyond group boundaries, to identify similarities between various groups, or to value cultural diversity (Thijs & Verkuyten, 2014) and should therefore be negatively related to prejudice.

Indeed, research showed that multicultural education is associated with more positive out-group evaluations among minority and majority youth (van Bommel et al., 2020). Previous findings further indicate that multicultural education is more frequently applied in culturally diverse schools compared to culturally homogeneous settings (Thijs & Verkuyten, 2014). Culturally diverse schools do not only increase the salience of intergroup relations but also offer more opportunities for positive intergroup contact (Allport, 1954). As such, they might also provide more options to directly implement norms of tolerance and respect than culturally homogeneous school contexts (Verkuyten & Thijs, 2013). However, despite its potential to reduce prejudice, emphasizing cultural differences may also increase the likelihood that young people will place individuals into rigid categories, thereby promoting stereotypes (Levy & Hughes, 2009). Although, the empirical evidence of multicultural education’s positive effects seems to outweigh potential negative consequences, scholars have called for more studies accounting for background characteristics, such as the level of cultural diversity at the national, regional, or school level to better understand its workings (Verkuyten & Thijs, 2013).

### School and Classroom Climate and Youth’s Intergroup Attitudes

Schools bring together young people from various social and cultural backgrounds and according to the contact hypothesis (Allport, 1954), intergroup contact should reduce prejudice. Therefore, the level of cultural diversity in schools or classrooms has been regarded as a very prominent predictor of intergroup attitudes and relations (van Geel & Vedder, 2011). Although findings on the direct effects of school or classroom diversity are not completely unambiguous, they point to beneficial outcomes (for a review, see Thijs & Verkuyten, 2014). The effects of diversity depend, however, also on the prevailing conditions within the classroom or school context. Following Allport’s contact hypothesis (Allport, 1954), a climate of support and cooperation among students (i.e., peer relationship climate) can challenge negative stereotypes, facilitate cross-ethnic friendship formation, and provide optimal conditions for positive intergroup contact, thereby amplifying its positive effects on intergroup attitudes (Tropp & Prenovost, 2008). Yet, even in culturally homogeneous classroom or school contexts, a good peer relationship climate can contribute to more positive intergroup evaluations. According to social learning perspectives (e.g., Bandura, 1977), schools are microlevel societies and experiencing supportive relationships with peers can serve as a template for interactions with other people in and outside of school (Dessel, 2010). Research on the effects of a supportive peer relationship climate in school on intergroup attitudes is scarce and offers mixed results. While positive effects of cooperative relationships in class on attitudes toward immigrants were reported in a longitudinal study among Swedish youth (Miklikowska et al., 2021), no significant associations were found among a sample of German adolescents (Gniewosz & Noack, 2008).

Apart from the peer relationship climate, another relevant - and related - characteristic of the school context is the prevailing democratic climate with student–teacher relations at its core. Attending a school where teachers encourage open discussion and provide opportunities to participate in decision making processes supports young people in becoming active and responsible citizens (Eckstein & Noack, 2014). Again, in line with socialization and social learning perspectives (e.g., Bandura, 1977), schools allow for students to learn about social and political processes on a small scale and therefore a democratic climate has the potential to stimulate youth’s own political awareness (Over & McCall, 2018). As part of a democratic climate, students may also experience that people, while differing in their opinions, beliefs, and lifestyles, can still treat each other with respect and openness. Tolerant attitudes toward diverse social groups can thus be another outcome of this process. The positive impact of a democratic classroom climate on adolescents’ civic knowledge and engagement has been repeatedly demonstrated (Torney-Purta et al., 2001). There is also empirical evidence that a democratic classroom climate is associated with positive intergroup attitudes (e.g., Solhaug & Osler, 2018). It should be noted, however, that democratic climate covers a broad spectrum of school experiences and, accordingly, has mostly been operationalized through various distinctive facets, such as open classroom climate for discussion (e.g., Carrasco & Torres Irribarra, 2018), fairness of teachers (e.g., Miklikowska et al., 2019), or opportunities for participation in decision making processes (e.g., Higdon, 2015).

Taken together, apart from its ethnic composition, the school context offers a variety of factors potentially relevant to the development of adolescents’ intergroup attitudes, such as school curriculum and school/classroom climate. While most research in this field is based on US and European samples, there are also large-scale assessments which allow for the consideration of school effects across various national contexts (e.g., International Civic and Citizenship Education Study, IEA ICCS; Schulz et al., 2018). Yet, studies employing longitudinal and multilevel designs are still rare.

### Age-Related Trends in the Effects of School Experiences

Young people spend a very long period of time in school ranging from childhood to late adolescence. So far, however, the question of whether the effects of school experiences differ according to students’ age remains largely unanswered. While experiences in school reach young people at a period in life that is generally considered to be of high relevance for the development of political attitudes and behaviors (Blakemore & Mills, 2014), intergroup attitudes—particularly involving visible social categories such as cultural background or gender—were found to consolidate and stabilize early in life (see Barrett & Oppenheimer, 2011 for a theoretical overview). Accordingly, attitudes toward immigrants were also shown to be well established already in early years and to increasingly stabilize throughout adolescence (Crocetti et al., 2021). Since once consolidated attitudes are less responsive to contextual influences, it may therefore be assumed that the effects of school experiences are less pronounced in older than in younger students (i.e., attitude consolidation hypothesis).

Alternatively, following the assumptions of motivational theories (e.g., Deci & Ryan, 1985), adolescents’ personal needs change over time. According to the stage-environment fit theory (Eccles & Midgley, 1989), the needs for autonomy, competence, and social relatedness increase throughout the adolescent years. In order to reach young people, schools should therefore account for these altering needs. Experiencing a school context that allows to engage in participatory-democratic principles (i.e., need for autonomy), to build strong relationships with peers (i.e., need for social relatedness), and to learn about cultural diversity (i.e., need for competence), might therefore be of higher relevance to older students than to younger students. As a consequence, it may also be assumed that older students are more susceptible to stimulating school experiences than younger students as they meet their altering needs (i.e., environment fit hypothesis). In addition, being exposed to characteristics of the school context for a longer period of time might also result in stronger effects of these factors in older students than in younger students. School experiences might then intensify over time. Applied to the area of cross-cultural friendships, for example, it could be shown that time spent together is substantially associated with positive intergroup attitudes (Davies et al., 2011).

### Individual and Collective Perceptions of School Experiences

Due to its multilevel nature, there are different perspectives on the school context. Although they might be biased, individual perceptions have been considered to be one crucial indicator. People react to their environment depending on how they perceive it, and therefore the significance of individual perceptions has been stressed in early sociological (Thomas & Thomas, 1928) and later social cognition research (Bodenhausen & Morales, 2012). However, individual perceptions may also vary systematically between students from different classrooms or schools. Students from the same school environment are exposed to the same routines, processes, and characteristics, which may contribute to certain dynamics and facilitate shared interpretations at the contextual level (Konishi et al., 2017). Disentangling individual level from classroom or school level effects can thus provide a more comprehensive understanding of schools’ workings, as processes may operate differently at different levels (Marsh et al., 2012). This is also important from a methodological and practical point of view. Methodologically, one and the same construct might have specific psychometric properties and meanings depending on the level of analysis (Lüdtke et al., 2009). Finally, accounting for individual and contextual processes has practical significance, as with tight schedules and a high diversity of students’ individual characteristics, it is difficult for teachers and educational staff to reach every single student in class. Thus, in order to provide guidelines for scalable interventions, it is important to gain a better understanding of processes operating not only at the individual but also at the classroom or school level.

### Background Information on the National and Regional Context

In line with contextual models of human development (e.g., Bronfenbrenner, 1979), the development of intergroup attitudes cannot be completely understood independently from macrocontextual characteristics. While school or classroom diversity has been considered a prominent predictor of intergroup attitudes at the school or classroom level (Thijs & Verkuyten, 2014), it also reflects processes at the broader societal level. Each country is characterized by a unique history of immigration. In Germany, this history varies considerably between regions and especially between the federal states in the Western and Eastern part (i.e., former German Democratic Republic, GDR) of the country. The present research is based on data that were collected in the federal state of Thuringia, historically a culturally rather homogenous region located in the Eastern part of Germany. Despite a steep increase of the immigrant population (i.e., people who immigrated to Germany themselves or have at least one parent who migrated to Germany) during the last decade (2010–2020), only around 7% of the total population of Thuringia is of immigrant descent (compared to 27% at the national level; Thüringer Ministerium für Migration, Justiz und Verbraucherschutz, TMMJV, 2019). The largest share of people of immigrant descent in Thuringia comes from Eastern European countries (e.g., Poland, Ukraine) and the former Soviet Union. Since 2015, refugees from crisis regions (e.g., Syria) represent an increasingly significant group (TMMJV, 2019). Although the proportion of people without an educational degree is higher among people of immigrant descent compared with people without immigrant background, educational inequalities between people with and without immigrant background were nonetheless found to be less pronounced in Thuringia than in other federal states of Germany (TMMJV, 2019).

Despite the low degree of cultural diversity within this region, national surveys repeatedly revealed substantial amounts of prejudice and intolerance toward immigrants (Reiser et al., 2018). This has, among others, been explained in terms of fewer opportunities for direct contact with people of varying cultural backgrounds (Pfister, 2018). Correspondingly, respondents from the Eastern part of Germany were found to have fewer cross-cultural friendships than respondents from the Western part (Zick et al., 2019). In the absence of cultural diversity, yet prevalent negative sentiments toward immigrants, the school context may play a particularly important role for youth’s attitudes as it can help students to reflect on privileges of the cultural majority and challenge prejudice (Swalwell, 2012).

## The Present Study

Although the empirical literature on school effects on youth’s attitudes toward immigrants is growing, to date there are still few longitudinal studies that also distinguished between individual- and contextual-level effects. Moreover, previous studies have rarely considered age-related susceptibility to school experiences. Drawing on longitudinal multilevel data from Germany, the present study aimed to address these gaps in the literature. As part of the study’s design, students who were either in 6th, 8th, or 10th grade at the first measurement point were surveyed over a period of 3 years. This allowed considering the effects of school experiences over a span of several years, ranging from 6th grade to 12th grade, therewith covering almost the entire period of secondary education in Germany. Within the multilevel framework, classrooms were chosen as unit of analysis at the contextual level, since students spent most of their time in class and generally remained in the same class over time. Microlevel dynamics within class were therefore expected to significantly shape students’ school experiences.

The first research question examined the effects of perceived multicultural education, supportive peer relationships in class, and democratic classroom climate on youth’s negative attitudes toward immigrants across time at the individual level (Level 1) and at the classroom level (Level 2). In line with socialization and social learning perspectives (e.g., Bandura, 1977), all three school characteristics were expected to be associated with less negative attitudes toward immigrants. Due to conceptual considerations underscoring the role of individual perceptions as well as higher-level dynamics, associations were expected both at the individual and classroom level.

The second research question addressed age-specific trends in the effects of school experiences. Based on theoretical as well as empirical findings both stronger effects among younger students (attitude consolidation hypothesis) and older students (environment fit hypothesis) may be expected. Therefore, an explorative approach was chosen which did not further specify assumptions concerning the age-related pattern of effects. This also applied to the level of analysis and thus the question whether age-specific patterns may primarily affect individual or classroom level processes.

## Method

### Sample

The present study was based on data from a comprehensive longitudinal project on adolescents’ civic development in the federal state of Thuringia in Germany (Noack, 2005). The sample consisted of 1292 adolescents who were surveyed over a period of three measurement points, each approximately 1 year apart (2003–2005). Adolescents’ age at the first measurement point (T1) was 13.86 years (*SD* = 1.45, age range: 12–18 years). Students came from different grade levels and were either in 6th (*n* = 394, 30.5%), 8th (*n* = 435, 33.6%), or 10th grade (*n* = 463, 35.9%) at T1. Students’ average age in the youngest cohort (i.e., 6th grade) was 12.29 years at T1 (*M*_age__T2 _= 13.25, *M*_ageT3 _= 14.37). Students’ average age in the middle aged cohort (i.e., 8th grade) was 13.48 years at T1 (*M*_ageT2 _= 14.50, *M*_ageT3 _= 15.46), and students’ age in the oldest cohort (i.e., 10th grade) was 15.52 at T1 (*M*_age__T2_ = 16.41, *M*_ageT3_ = 17.43). Gender was roughly equally distributed (*n*_female_ = 669, 51.8%). Only a few students reported not having German citizenship (*n* = 16, 1.2%). This low proportion is characteristic of this region in Germany (TMMJV, 2019). All participating schools were randomly selected from two major school tracks—a higher, college-bound track (*Gymnasium*) leading to the qualification exams to enter university (i.e., *Abitur*) after grade 12, and a lower, more practically oriented track (*Regelschule*) designed to lead to apprenticeship-based vocational training after grade 10. Altogether, 73 classes from 36 higher and lower track schools were included in the study, whereby the number of participating schools was equally distributed across both school types. The number of students per class varied between 6 and 28 (*M* = 17.69, *SD* = 5.79). Slightly more students attended the college-bound track (*n* = 760, 58.8%) than the practically oriented track (*n* = 532, 41.2%).

The procedure of data collection was similar for all schools: Once schools had agreed to participate, students and parents were asked for their consent. Finally, the survey was conducted during an extra-curricular lesson in class. Students completed a questionnaire on different civic topics, which took approximately 90 min. During this time a research assistant from the project was present in class to administer the survey. After each measurement point, classrooms received a small contribution to the class fund.

### Measures

If not indicated differently, the response options ranged from 1 = I do not agree at all to 4 = I totally agree. A complete summary of the scales’ item wordings is provided in the online supplemental material (Supplementary Table S1).

#### Negative attitudes toward immigrants

Attitudes toward immigrants were assessed with six items (e.g., “Immigrants take away our jobs”; Balke et al., 2002; Dicke et al., 2000; Kracke & Held, 1994). Cronbach’s alpha coefficients indicated good internal consistencies (*α*_T1 _= 0.83, *α*_T2 _= 0.86, *α*_T3 _= 0.86).

#### School Experiences

School experiences were assessed by three indicators covering different facets of the school environment at Time 1. Perceived Multicultural Education was assessed with a single item (“Some teachers try very hard to familiarize us with the culture and points of view in other countries”). Supportive Peer Relations in class were assessed with four items (e.g., “There is a strong sense of community in our class”; *α*_T1_ = 0.64; Eder, 1998). Democratic Classroom Climate was measured by a 7-item-scale that included questions referring to open classroom climate for discussion, teachers’ fairness, and participation in decisions (e.g., “Students are encouraged to make up their minds about issues”; Eder, 1998; Torney-Purta et al., 2001; *α*_T1_ = 0.77). Besides students’ individual ratings, class-average ratings of all three school experiences were obtained by aggregating students’ individual responses at the classroom level (see Analytical Procedure).

#### Covariates

Age (in years), Gender (0 = male, 1 = female), SES (i.e., level of parental education; 1 = no degree, 2 = finishing school after 8th grade, 3 = finishing school after 10th grade, 4 = Abitur/finishing school after 12th grade, 5 = university degree), Citizenship (0 = non-German, 1 = German) and School Track (0 = lower track, 1 = higher track) served as covariates. Grade level (age cohort; i.e., 6th, 8th, 10th grade at T1) was furthermore considered as moderator variable.

### Attrition Analysis

As in most longitudinal studies, not all students continued to participate in the survey throughout the years (complete data: *n* = 583, 45.1% of initial sample). Overall, two patterns of missingness emerged: 458 students (35.4%) were missing only at one wave, while 251 (19.4%) were missing at two waves. Little’s MCAR test (Little, 1988), which included all study variables, was significant (*χ*^2^[318] = 318.23, *p* < 0.001) suggesting that data were not missing completely at random. To gain a deepened understanding, follow-up analyses were run in which adolescents with no missing data were compared to adolescents who did not participate at one or more measurement points (*n* = 709, 54.9%). At Time 1 students with and without missing data did not differ in their negative attitudes toward immigrants (*t*[1290] = 1.176, *p* = 0.240). Likewise, analyses yielded no significant differences concerning gender (*χ*^2^[1, *N* = 1292] = 1.377, *p* = 0.214), SES (*t*[1280] = 1.334, *p* = 0.180), or citizenship status (*t*[1282] = 0.378, *p* = 0.706). Yet, lower track students had significantly more missing data than higher track students (*χ*^2^[1, *N* = 1292] = 25.050, *p* < 0.001) and older students had significantly more missing data than younger students (*t*[1290] = 15.381, *p* < 0.001). These two patterns can be explained by the study’s design. Since lower track students left school after 10th grade, they could no longer be reached in school for participation at the later measurement points, which caused a systematic drop-out among older and lower track students. In order to prevent a further reduction of the initial sample size, missingness was addressed using a full information maximum likelihood approach (maximum likelihood estimation with robust standard errors, MLR; see, e.g., Jeličič et al., 2009).

### Analytic Procedure

To account for the hierarchical nature of the data with students being nested within classrooms (and schools), multilevel modeling was used. In doing so, classrooms were chosen as the unit of analysis at the contextual level. Students spent most of their time in classrooms and primary analyses showed that the proportion of variance at the classroom level was comparable—if not greater—than the amount of variance located at the school level^1^.

Two-Level Growth Curve Modeling with manifest indicators was employed using Mplus 8.6 (Muthèn & Muthèn, 1998-2017) to examine changes in youth’s negative attitudes toward immigrants at the individual level (Level 1) and classroom level (Level 2) across time (T1–T3). As part of the model specification, the intercept factor was defined as the initial status at Time 1 (i.e., latent mean at T1). The slope factor describes the amount of linear change in youth’s negative attitudes toward immigrants from one measurement point to another.

In a first step, an unconditional model was specified to depict average trajectories in the entire sample (Model 1). In a second step, school experiences were added to the analyses at the individual and classroom level (Model 2.1–2.3), while also controlling for the effects of significant covariates (Model 3.1–3.3). The effects of school experiences on the intercept and slope factor were examined simultaneously at the individual and at the classroom level. In order to do so, individual ratings were aggregated at the classroom level whereby the resulting mean is considered as indicator of collective perceptions at Level 2 (Lüdtke et al., 2009). Since Mplus offers a latent aggregation of Level 1 predictors, the aggregated Level 2 indicators are treated as latent variables (i.e., multilevel latent covariate model; Lüdtke et al., 2008). This approach has shown to correct for unreliability of Level 2 indicators and to lead to less biased parameter estimates (Lüdtke et al., 2009). In the case of significant Level 1 and Level 2 effects, it was further assessed whether the relationship found at the classroom level was significantly different from the relationship at the individual level (i.e., contextual effect; difference between Level 1 and Level 2 effect; Raudenbush & Bryk, 2002). For models including covariates (Model 3.1–3.3), Level 1 covariates (i.e., age, gender, SES, citizenship) were centered at their grand mean, since this centering option allows for accounting for covariate effects at Level 1 and Level 2 (Lüdtke et al., 2009).

In a third step, it was examined whether the effects of school experiences on negative attitudes toward immigrants would differ according to students’ age. Since in multilevel modeling, higher-level variables are usually conceptualized as moderator (e.g., Aguinis et al., 2013) and since students of the same age group attended the same grade level (6th, 8th, 10th grade at T1)^2^, the latter was chosen as moderator variable. This allowed taking the data’s age-based grouping further into account. Due to its categorical scaling, grade level was dummy coded, whereby the youngest age group (6th grade at T1) served as reference category. Two sets of moderation analyses were carried out: (1) To test whether grade level (Level 2 variable) would moderate the association between individual perceptions of school experiences and negative attitudes toward immigrants (Level 1 relationship), cross-level interactions were specified (via random-slope multilevel modeling; Model 4.1–4.3). (2) To test for interactions between class-average perceptions of school experiences and grade level, interaction terms between each school variable and the dummy coded grade level indicators were created and added as predictors at Level 2 (Model 5.1–5.3).

Maximum likelihood estimation with robust standard errors (MLR) was used for all analyses, since it has been shown to be robust against deviations from statistical assumptions (Field & Wilcox, 2017). Finally, model fit was evaluated based on established fit indices (Hu & Bentler, 1999)^3^. Unless indicated differently, the estimated models fit the data well.

## Results

### Preliminary Analyses

#### Zero-order correlations

Table 1 summarizes zero-order correlations at Level 1 and Level 2 for the main study variables. At the individual level, supportive peer relations in class and democratic classroom climate (at T2 and T3) were negatively related with attitudes toward immigrants. At the classroom level, only supportive peer relations in class showed a significant association with negative attitudes toward immigrants at T1.

**Table 1:** Zero-order correlations and intraclass correlations of main study variables

Variables		*M*	SD	1	2	3	4	5	6
1	Anti-Immigrant Attitudes T1	2.51	0.74	*0.15/0.76*	0.77**	0.60**	–0.18	–0.29*	0.03
2	Anti-Immigrant Attitudes T2	2.55	0.75	0.63**	*0.12/0.70*	0.67**	–0.13	0.21	0.02
3	Anti-Immigrant Attitudes T3	2.52	0.72	0.55**	0.67**	*0.10/0.67*	–0.19	–0.25	0.12
4	Dem. Classroom Climate	2.88	0.46	–0.02	–0.06*	–0.11**	*0.12/0.70*	0.79**	0.53**
5	Supportive Relations in Class	2.87	0.55	–0.06*	–0.06*	–0.09*	0.40**	*0.15/0.75*	0.35
6	Multicultural Education	2.73	0.75	0.02	0.02	0.04	0.36**	0.21**	*0.05/0.48*

Correlations at the individual level (Level 1) are displayed below the diagonal, correlations at the classroom level (Level 2) are displayed above the diagonal, ICC(1)/ICC(2) are presented in italic font within the diagonals. *N*_L1_ = 1292 students, *N*_L2_ = 73 classrooms with average size of 17.69 students/class

*p < 0.05, **p < 0.01

#### Psychometric quality of classroom-level constructs

To determine whether the aggregation of individual ratings resulted in reliable indicators of higher-level processes, intraclass correlation coefficients (ICC) were estimated. While ICC(1) describes the amount of variation between classrooms, ICC(2) assesses the reliability of class-level means (as indicator of the “true” classroom mean; Lüdtke et al., 2009). The results are summarized in Table 1. Except for perceived multicultural education, ICC(1)s were larger than 0.10, indicating that more than 10% of the variance for negative attitudes toward immigrants, supportive peer relations in class, and democratic classroom climate was located at the classroom level, supporting the adoption of a multilevel perspective to the research questions (Julian, 2001). Estimations of ICC(2) for negative attitudes toward immigrants were 0.76 at T1, 0.70 at T2, and 0.67 at T3 and, thus, above or close to the recommended cut-off value of 0.70 (Bliese, 1998). While values of ICC(2) were also adequate for supportive peer relationships (0.76) and democratic classroom climate (0.70), the value was considerably lower for perceived multicultural education (0.48).

### Main Analyses

#### Effects of school experiences on negative attitudes toward immigrants

At first, a baseline model was specified to depict average changes in youth’s negative attitudes toward immigrants (Model 1; *χ*^2^[1,*N* = 1192] = 0.68, *p* = 0.411, CFI = 1.000, TLI = 1.000, RMSEA = 0.000, SRMR_Within_ = 0.000, SRMR_Between _= 0.007). The results showed no significant mean-level changes across time, as indicated by the non-significant slope mean (*B* = 0.003, *SE* = 0.015, *p* = 0.830). Yet, at the individual level, both the intercept (σ^2^ = 0.316, *SE* = 0.033, *p* < 0.001) and the slope variance (σ^2^ = 0.053, *SE* = 0.014, *p* < 0.001) were significantly different from zero pointing to interindividual differences at T1 and in changes across time. While there was also significant variation around the intercept factor at the classroom level (σ^2^ = 0.078, *SE* = 0.019, *p* < 0.001), no significant variation was found around the slope factor (σ^2^ = 0.003, *SE* = 0.008, *p* = 0.669), indicating that there were no significant differences between classrooms in their rate of change. Due to this lack of variation, the slope variance was constrained to zero, which did not significantly affect model fit [Δ*χ*^2^(2) = 2.15, *p* = 0.341]^4^. Effects on the slope factor at the classroom level were therefore not further considered in subsequent analyses^5^.

Next, school experiences were added to the analyses to predict the intercept and slope factor at the individual and classroom level. In doing so, each school indicator was considered separately for its predictive value (Models 2.1–2.3; for fit indices, see Supplementary Table S2). Table 2 provides a summary of the findings. No significant effects on the intercept or slope factor were found for perceived multicultural education—neither at the individual nor at the classroom level. While supportive peer relations in class also showed no significant effect on the intercept or slope factor at the individual level, there was a significant effect on the intercept factor at the classroom level (*β* = –0.405, *SE* = 0.133, *p* = 0.002). This effect was significantly stronger than the Level 1 effect (contextual effect: *B* = –0.461, *SE* = 0.174, *p* = 0.008), indicating that irrespective of whether students personally perceived relations to peers in class to be supportive, they reported fewer negative attitudes if they were in classrooms which were on average characterized by high levels of supportive peer relations (see Table 2). And finally, while the results at the individual level for democratic classroom climate also revealed no significant effect on the intercept factor, there was a significant effect on the slope factor (*β* = –0.119, *SE* = 0.050, *p* = 0.017), indicating that youth who perceived the classroom climate as more democratic at T1 reported a decline in negative attitudes toward immigrants across time compared to youth who perceived the classroom climate to be less democratic. Moreover, as was the case for supportive peer relations in class, there was a significant effect on the intercept factor at the classroom level (*β* = –0.271, *SE* = 0.135, *p* = 0.045). Again, this effect was significantly different from the effect at the individual level (contextual effect: *B* = 0.426, *SE* = 0.210, *p* = 0.043). Thus, irrespective of whether students personally perceived their classrooms to be democratic, they reported fewer negative attitudes toward immigrants if they came from classrooms in which students perceived the average climate to be democratic.

**Table 2:** Prediction of negative attitudes toward immigrants by school experiences

	Perceived multicultural education as predictor of attitudes				Supportive peer relationships as predictor of attitudes				Democratic classroom climate as predictor of attitudes			
	Model 2.1				Model 2.2				Model 2.3			
	Intercept		Slope		Intercept		Slope		Intercept		Slope	
*Level 1*	*B*	*SE*	*B*	*SE*	*B*	*SE*	*B*	*SE*	*B*	*SE*	*B*	*SE*
School experience	0.02	0.03	0.01	0.02	–0.02	0.05	–0.01	0.03	–0.00	0.05	–0.06*	0.03
*R*^2^	0.00	0.00	0.00	0.00	0.00	0.00	0.00	0.00	0.00	0.00	0.02	0.01
*Level 2*												
School experience	–0.05	0.27	–	–	–0.48**	0.17	–	–	–0.43*	0.21	–	–
*R*^2^	0.00	0.00	–	–	0.16	0.11	–	–	0.07	0.07	–	–
	Model 3.1				Model 3.2				Model 3.3			
	Intercept		Slope		Intercept		Slope		Intercept		Slope	
*Level 1*	*B*	*SE*	*B*	*SE*	*B*	*SE*	*B*	*SE*	*B*	*SE*	*B*	*SE*
School experience	0.01	0.03	0.01	0.02	–0.03	0.05	–0.01	0.02	–0.02	0.04	–0.06*	0.03
Age	–0.03	0.02	–	–	–0.03	0.02	–	–	–0.03	0.02	–	–
Gender	–0.09*	0.04	–	–	–0.09	0.05	–	–	–0.09*	0.04	–	–
SES	–0.07**	0.03	–	–	–0.07**	0.03	–	–	–0.07**	0.03	–	–
Citizenship	0.77**	0.08	–	–	0.81**	0.09	–	–	0.81**	0.08	–	–
*R*^2^	0.05**	0.02	0.00	0.00	0.05**	0.01	0.00	0.00	0.05**	0.01	0.02	0.01
*Level 2*												
School experience	–0.84**	0.31	–	–	–0.37*	0.16	–	–	–0.56**	0.32	–	–
School track	–0.40**	0.06	–	–	–0.29**	0.06	–	–	–0.32**	0.05	–	–
*R*^2^	0.83**	0.17	–	–	0.63**	0.11	–	–	0.68**	0.10	–	–

*B* unstandardized parameter estimate, *SE* standard error

Level 1 = individual level, Level 2 = classroom level. *N*_L1_ = 1292 students, *N*_L2_ = 73 classrooms

*p < 0.05, **p < 0.01

In a next step, the models were repeated while also controlling for significant covariate effects^6^ (i.e., age, gender, citizenship, and SES on the intercept at Level 1 and school track on the intercept at Level 2; Models 3.1–3.3; for fit indices, see Supplementary Table S2). The findings are also summarized in Table 2. Whereas the results at the individual level remained the same after controlling for covariate effects, the result pattern changed at the classroom level. In addition to supportive peer relations in class and democratic classroom climate, perceived multicultural education also showed a significant effect on the intercept factor at Level 2 (*β* = –0.572, *SE* = 0.193, *p* = 0.003, see Table 2 for unstandardized parameter estimates). Accordingly, when taking age, gender, citizenship, SES, and school track differences into account, students from classrooms in which teachers were perceived to explicitly address cultural issues reported fewer negative attitudes toward immigrants than students from classrooms in which cultural topics were not explicitly addressed. A follow-up analysis showed that the significant association between multicultural education and negative attitudes toward immigrants at the classroom level was only present among male students from higher track schools.^7^

#### Grade level as moderator of the effects of school experiences

In a last step, it was examined whether the effects of school experiences on negative attitudes toward immigrants differed according to age (i.e., grade level). To do so, a further set of two-level growth curve models was conducted, which included the cross-level interactions examining whether the effect of students’ individual perceptions of school experiences on the intercept or slope factor at Level 1 different according to grade level (i.e., dummy coded Level 2 indicator, Model 4.1–4.3). As in all previous model specifications, the classroom level effects of school experiences on the intercept at Level 2 were also taken into account. The results showed that neither the effects of perceived multicultural education, nor supportive peer relations in class, or democratic classroom climate at Level 1, differed according to grade level. Table 3 summarizes the main model coefficients.

**Table 3:** Moderation analyses examining effects of school experiences at Level 1 on negative attitudes toward immigrants according to age group (grade level)

	Perceived multicultural education as predictor of attitudes				Supportive peer relationships as predictor of attitudes				Democratic classroom climate as predictor of attitudes			
	Model 4.1				Model 4.2				Model 4.3			
	Intercept		Slope		Intercept		Slope		Intercept		Slope	
*Level 1*	*B*	*SE*	*B*	*SE*	*B*	*SE*	*B*	*SE*	*B*	*SE*	*B*	*SE*
School experience	0.04	0.06	–0.03	0.03	0.00	0.08	–0.01	0.04	–0.06	0.08	–0.09*	0.04
*Level 2*												
Constant	2.41	0.42	–	–	3.72	0.38	–	–	3.39	0.48	–	–
School experience	–0.04	0.14	–	–	–0.40**	0.12	–	–	–0.29	0.16	–	–
Dummy_Grade 8	0.10	0.08	–	–	0.04	0.08	–	–	0.05	0.09	–	–
Dummy_Grade 10	–0.07	0.09	–	–	–0.13	0.07	–	–	–0.12	0.08	–	–
*Cross-level Interaction*												
School experience × Dummy_Grade 8	–0.04	0.07	0.06	0.04	–0.10	0.10	0.03	0.06	–0.13	0.11	0.07	0.06
School experience × Dummy_Grade 10	–0.02	0.08	0.06	0.04	0.01	0.12	0.02	0.06	–0.06	0.13	0.07	0.06
*Additional Information*												
σ² Level 1	0.32**	0.03	0.06**	0.01	0.31**	0.03	0.05**	0.01	0.31**	0.03	0.05**	0.01
σ² Level 2	0.06**	0.01	–	–	0.05**	0.01	–	–	0.06**	0.01	–	–
AIC	5357.792				5520.540				5520.191			
BIC	5485.884				5649.393				5649.134			

*B* unstandardized parameter estimate, *SE* standard error, *AIC* Akaike information criterion, *BIC* Bayesian information criterion

Level 1 = individual level, Level 2 = classroom level. Dummy_Grade 8 (0 = other, 1 = grade 8), Dummy_Grade 10 (0 = other, 1 = grade 10)

*p < 0.05, **p < 0.01

**Fig. 1:**
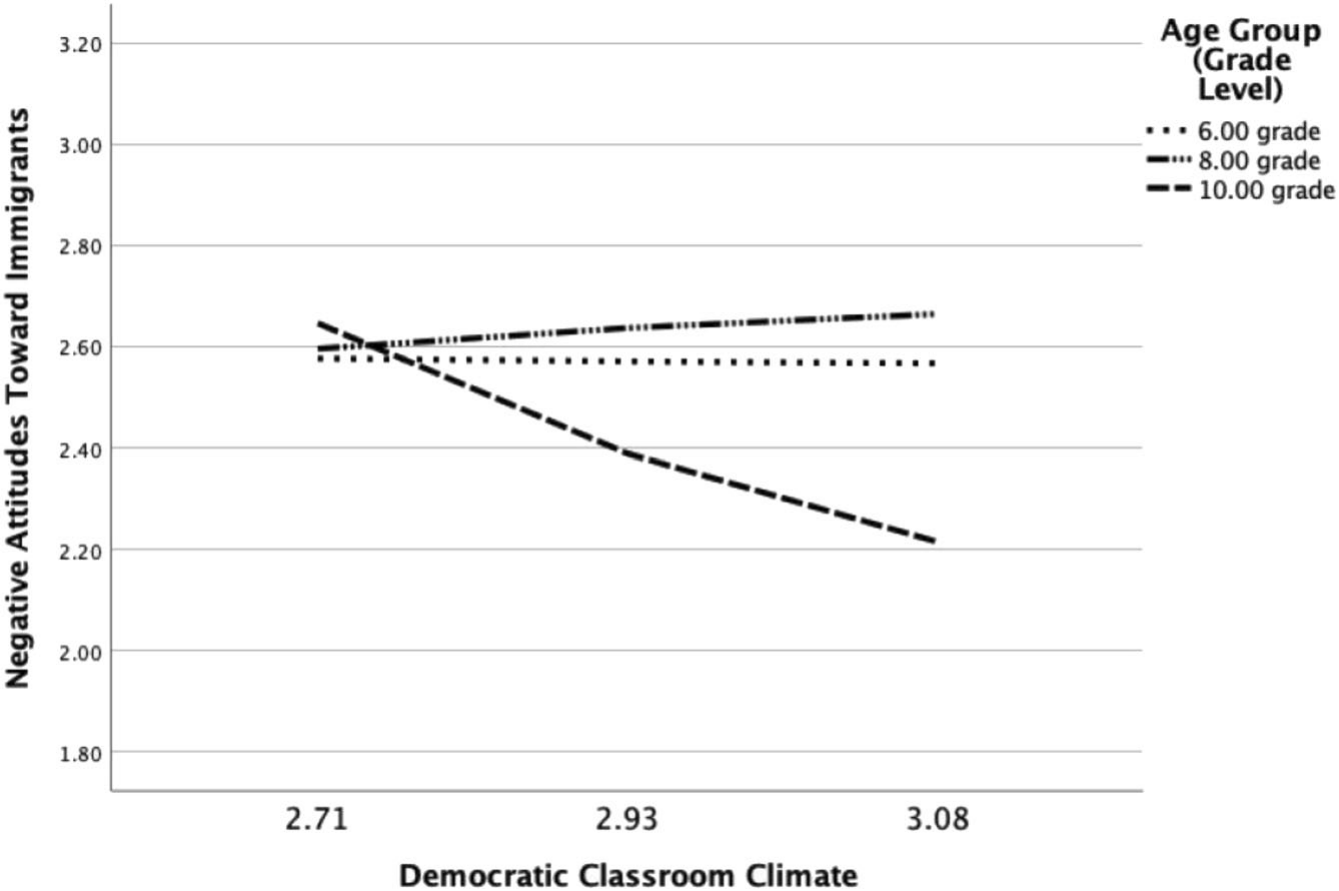
Association between democratic classroom climate at Level 2 and attitudes toward immigrants at according to age group (grade level)

Subsequently, age-specific patterns were tested for all classroom level effects (Model 5.1–5.3). As part of the model specification, the intercept factor at Level 2 was predicted by school experience, grade level indicators (dummy coded), and the interaction terms between school experience and grade level. The effects of school experiences were also included at Level 1 (on the intercept and slope factor). The results showed that class-average perceptions of perceived multicultural education and supportive peer relationships did not vary by grade level. Yet, grade level moderated the Level 2 effect of democratic classroom climate (see Table 4). A follow-up analysis in which the effects were considered separately according to grade level showed that class-average perceptions of a democratic classroom climate were significantly associated with fewer negative attitudes toward immigrants at T1 among 10th grade students (*β* = –0.682, *SE* = 0.210, *p* = 0.001), while the effects were not significant among students from 6th and 8th grade (grade 6: *β* = 0.062, *SE* = 0.151, *p* = 0.682; grade 8: *β* = 0.060, *SE* = 0.270, *p* = 0.823). The interaction is also displayed in Fig. 1. It should be noted that a similar tendency emerged for supportive peer relationships in class, although the interactions with the dummy coded grade level indicators did not reach significance (associations between supportive peer relationships at Level 2 and negative attitudes toward immigrants according to grade level; grade 6: *β* = –0.175, *SE* = 0.180, *p* = 0.331; grade 8: *β* = –0.194, *SE* = 0.266, *p* = 0.466; grade 10: *β* = –0.626, *SE* = 0.286, *p* = 0.028). The associations between perceived multicultural education at Level 2 and negative attitudes toward immigrants were not significant for all grade levels (grade 6: *β* = 0.114, *SE* = 0.146, *p* = 0.436; grade 8: *β* = 0.165, *SE* = 0.347, *p* = 0.633; grade 10: *β* = –0.134, *SE* = 0.271, *p* = 0.621).

**Table 4:** Moderation analyses examining effects of school experiences at Level 2 on negative attitudes toward immigrants according to age group (grade level)

	Perceived multicultural education as predictor of attitudes				Supportive peer relationships as predictor of attitudes				Democratic classroom climate as predictor of attitudes			
	Model 5.1				Model 5.2				Model 5.3			
	Intercept		Slope		Intercept		Slope		Intercept		Slope	
*Level 1*	*B*	*SE*	*B*	*SE*	*B*	*SE*	*B*	*SE*	*B*	*SE*	*B*	*SE*
School experience	0.02	0.03	0.01	0.02	–0.03	0.05	–0.01	0.03	–0.00	0.05	–0.06*	0.03
*Level 2*												
Constant	2.27	0.51	–	–	2.73	0.75	–	–	2.37	0.79	–	–
School experience	0.09	0.18	–	–	–0.14	0.24	–	–	0.06	0.25	–	–
Dummy_Grade 8	–0.04	0.85	–	–	0.26	1.01	–	–	0.14	1.05	–	–
Dummy_Grade 10	1.00	0.87	–	–	1.37	0.92	–	–	2.85	0.97	–	–
School experience × Dummy_Grade 8	0.06	0.30	–	–	–0.07	0.34	–	–	–0.01	0.36	–	–
School experience × Dummy_Grade 10	–0.40	0.32	–	–	–0.51	0.30	–	–	–1.01**	0.32	–	–

*B* unstandardized parameter estimate, *SE* standard error

Level 1 = individual level, Level 2 = classroom level. Dummy_Grade 8 (0 = other, 1 = grade 8), Dummy_Grade 10 (0 = other, 1 = grade 10)

*p < 0.05, **p < 0.01

#### Sensitivity analyses

Excluding classrooms with less than 10 students (*n*_classrooms _= 7, *n*_students_ = 56) or adding grade level as an additional covariate at Level 2 (in Model 3.1–3.3) did not affect the overall pattern of findings. The results were also replicated when all school experiences were examined simultaneously in one model. The only exceptions were the Level 2 effects of democratic classroom climate and supportive peer relations in class. Both variables were highly correlated at the classroom level (*r* = 0.79, see Table 1) and therefore confounded. After accounting for this, however, the overall pattern of results could be replicated.

## Discussion

With its goal to educate tolerant and mature citizens, schools have been described as an important socialization context in youth (Neundorf & Smets, 2017). While school experiences were shown to be related to youth’s intergroup attitudes (e.g., Barber et al., 2013), the empirical evidence for that is still limited, especially with respect to longitudinal designs. Moreover, although school experiences accompany young people from late childhood into late adolescence, age-specific effects have rarely been considered to date. The present 3-wave study from Germany aimed to contribute to the literature by accounting for the effects of perceived multicultural education, supportive peer relations in class, and democratic classroom climate on German youth’s negative attitudes toward immigrants. Due to the data’s multilevel and cohort-sequential nature, processes at the individual and classroom level could be compared over a wide age span ranging from 12 to18 years. Although the results revealed few effects at the individual level (i.e., only for democratic classroom climate), all three school indicators were cross-sectionally related to less negative attitudes toward immigrants at the classroom level (for perceived multicultural education, however, only after controlling for the effects of socio-demographic covariates). Moreover, age-related patterns were found for the effect of democratic climate at the classroom level, pointing to stronger effects among older than among younger students.

### School Experiences and Attitudes toward Immigrants

The results for the first research question can be summarized in three patterns: First, perceived multicultural education was not related to youth’s negative attitudes toward immigrants at the individual level (neither cross-sectionally at Time 1 nor longitudinally across time). This is not in line with previous research showing significant effects of multicultural education on intergroup attitudes (e.g., Verkuyten & Thijs, 2013) and might be related to the characteristics of the sample. Data were collected in a region of Germany where only around 7% of the population are of immigrant descent (TMMJV, 2019) and, consequently, the students in the current study experienced culturally homogeneous school environments. Therefore, questions about cultural diversity that usually arise from everyday interactions between class- or schoolmates may either not have developed at all or might have been personally less salient than in culturally heterogeneous school contexts. Consequently, individual perceptions of teachers’ handling of cultural topics might also be less relevant for youth’s intergroup attitudes. Indeed, research has shown that multicultural education is more frequently applied in culturally diverse than in homogeneous classrooms (Thijs & Verkuyten, 2014).

However, at the classroom level, the bivariate association between class-average perceptions of multicultural education and youth’s attitudes toward immigrants was significant for male students from higher track classrooms (see Footnote 7 and Supplementary Table S3). One possible explanation for the responsiveness of this particular subgroup might be that at the contextual level, higher track classrooms are characterized by a less negative climate toward immigrants than lower track classrooms (e.g., Schmid & Watermann, 2010), which could facilitate the discussion of cultural-related topics. In addition - and in line with previous research (Higdon, 2015)—male students reported more negative attitudes toward immigrants than female students. Like intergroup contact, multicultural education might be particularly beneficial for more prejudiced youth (e.g., Hodson & Dhont, 2015). Hence, while more prejudiced male students might be more responsive to classroom-level effects of multicultural education, it may require the additional impact of a non-prejudiced classroom environment for multicultural education to unfold its effects. To draw firm conclusions, however, a more nuanced investigation of the interplay between socio-demographic variables and dynamics within the classroom is needed.

Second, no significant effects emerged for supportive peer relations in class at the individual level (neither cross-sectionally nor longitudinally). The absence of a Level 1- effect might be attributed to the fact that the indicator explicitly addressed dynamics within class. Research showed more consistent associations at the individual level by using a measure that focused on personal feelings of social belonging in school (Gniewosz & Noack, 2008). Future studies could therefore compare the effects of individual- vs. classroom-oriented indicators of peer relations more systematically. However, there was a significant effect of supportive peer relations at the classroom level at Time 1. Thus, irrespective of how students personally perceived their relationships with classmates, there were certain dynamics at the classroom level that mattered. Theoretically, the classroom-level effect is in line with social learning and socialization perspectives (Bandura, 1977). Being surrounded by supportive peers seems to set an example of how to relate to other people inside or outside of school (Dessel, 2010). While this finding is consistent with previous research showing associations between cooperative peer relations and youth’s attitudes toward immigrants (Miklikowska et al., 2021), it further adds to these studies by showing that supportive relations with classmates do matter in culturally homogeneous school settings where opportunities for cross-cultural contact are limited.

Third, while students’ individual perception of a democratic classroom climate had no significant effect at Time 1, it predicted a decline in negative attitudes toward immigrants across time at the individual level. At the classroom level, class-average perceptions of a democratic classroom climate were also associated with less negative attitudes toward immigrants at Time 1. These results are generally consistent with previous studies using more narrow and specific indicators of democratic classroom climate (i.e., perceived teacher support and fairness (Miklikowska et al., 2019), open classroom climate for discussion (Carrasco & Torres Irribarra, 2018), or participation in decision making processes (Higdon, 2015). This study therefore replicates the effects found in existing literature with a broader indicator of democratic classroom climate. Together with previous research, and also in line with social learning and socialization perspectives (Bandura, 1977), these results suggest that experiencing a climate in which people can differ in their opinions and lifestyles, but still treat each other with respect contributes to the development of less prejudiced intergroup attitudes. However, the results also revealed different patterns at the individual and classroom level. Whereas a longitudinal effect was found at the individual level, the dynamics at the classroom level are reflected in a cross-sectional association (yet only among older students). The longitudinal effect at the individual level might be due to the fact that processes related to youth’s individual perception need time to unfold. Thus, perceiving a democratic climate may encourage adolescents to reflect on their own political positions and to compare them to the views and lifestyles of others. While this may eventually shape youth’s attitudes toward diverse groups, such school experiences are often not a matter of conscious decision. Therefore, students may need to personally experience a democratic school climate for some time for its effects to take hold. To better understand the underlying processes, future studies should compare potential mediating variables at the individual and at the classroom level in order to gain insight into the respective mechanism.

In sum, school experiences were found to be related to youth’s negative attitudes toward immigrants. At the same time, the findings underscore that both processes at the individual and contextual level should be considered, supporting the adoption of a multilevel perspective. The most consistent effects of school experiences were identified at the classroom level, which is important from a pedagogical and practical perspective. Tight curricula and a high diversity of students’ individual needs make it difficult for teachers to reach every single student in class. Therefore, a deepened understanding of processes operating at the classroom level could help to provide more general recommendations or guidelines for teachers. Although addressing unfavorable intergroup attitudes within school is a long and challenging task, it is the knowledge about underlying processes that provides a crucial starting point. Raising teachers’ and students’ awareness of the importance of social and democratic processes in class could be a first step. The integration of collaborative learning strategies, interactive and engaging classroom activities, or instructional methods that promote a dialog between teachers and students might then represent some practical and concrete examples of how to foster an open and supportive climate.

### Age-related Trends in the Effects of School Experiences

The second research question examined whether the effects of school experiences on youth’s negative attitudes toward immigrants would differ according to students’ age (i.e., grade level). While no indication of age-specific effects of school experiences at the individual level was found, the results showed that the dynamics at the classroom level differed by grade level. In particular, class-average perceptions of democratic classroom climate were only associated with less negative attitudes toward immigrants among older (10th grade) but not younger students (6th or 8th grade). This result is in line with the assumptions of the stage-environment fit theory (Eccles & Midgley, 1989). It suggests that a democratic classroom context may meet students’ growing needs for autonomy and efficacy. It might, however, also mean that a longer exposure to a democratic classroom dynamic is needed to observe its effects. Future studies should therefore examine the processes underlying age-specific patterns in greater detail.

Although there was also a tendency for the effect of supportive peer relations at the classroom level to be stronger among older than among younger students, the moderation by grade level did not reach significance. The attenuated age-related pattern might be explained by the fact that peer relations are—despite changes in structure and dynamics—of high significance throughout the adolescent years (Bowker & Ramsey, 2011). There was also no indication that the effect of perceived multicultural education on students’ negative attitudes toward immigrants differed by grade level. These results are not in line with the environment fit hypothesis. Students seem to have the necessary cognitive capacity to benefit from the existence of multicultural educational strategies at the outset of adolescence. Correspondingly, a meta-analytical overview found the effects of multicultural education on intergroup attitudes to be stronger among adolescent than among pre-adolescent students, but did not assume the effects to differ within the adolescent group (Okoye-Johnson, 2011).

In sum, age-related trends were identified for the classroom-level effect of democratic climate, which was only related to attitudes toward immigrants among older but not among younger students. This indicates that age matters, yet only for certain school indicators. Further research on age-related effects of school experiences is needed to draw more definite conclusions about the generalizability of these findings. Knowing about specific processes depending on students’ or schools’ contextual characteristics can help to provide more tailored advice for schools to create an inclusive environment and to develop strategies to reduce prejudice.

### Limitations and Future Research

Some limitations of this research need to be noted. As in most longitudinal studies, not all students participated at all measurement points. Attrition was particularly high among older and lower track students. To account for the potential impact of data attrition, missing values were taken into account in the model estimation. Although this is a highly recommended method to deal with missingness (Jeličič et al., 2009), a possible bias due to data attrition cannot be completely ruled out. Conceptually, it should be noted that school experiences were examined while drawing on indicators primarily reflecting classroom level processes (i.e., supportive peer relations in class, democratic classroom climate). Since the students in the present study spent most of their time within classrooms, these microlevel dynamics represent an important aspect of their school-based experiences. Yet, to get a more holistic understanding of contextual processes, future studies should further differentiate dynamics at the classroom and school level.

Several limitations concern the adopted measures. First, while the internal consistency of supportive peer relationships at Level 2 was adequate, it was only marginally acceptable at the individual level. To account for measurement error, latent measurement models could, for example, be specified at the individual level in future studies. Second, the measure of perceived multicultural education, which was based on a single-item indicator, poses another limitation (e.g., Loo, 2002). Reliability estimates of class-average ratings [i.e., ICC(2)] furthermore remained clearly below the recommended threshold. Besides these psychometric limitations, conceptually broader indicators should be applied in future studies. Apart from addressing cultural topics, educational strategies that foster students’ critical thinking or discuss discrimination and racism could be included as well (for an overview see Verkuyten & Thijs, 2013). Third, negative attitudes toward immigrants were assessed with an explicit measure and might therefore underlie a certain bias due to students’ external or internal motivation to respond without prejudice (Plant & Devine, 1998). Although similar indicators were used in previous studies (e.g., Miklikowska et al., 2021), more research comparing explicit and implicit measures of intergroup attitudes among adolescents is needed to gain a thorough understanding of processes causing and maintaining negative attitudes toward immigrants (see, for example, Ewoldsen, 2020).

Finally, several characteristics of the data set need to be pointed out: The study was conducted in the federal state of Thuringia in Germany, which is culturally a rather homogenous region (with currently approx. 7% of the population being of immigrant descent). Although this shows that school experiences matter for adolescents’ intergroup attitudes even in the absence of cultural diversity, research from other regions is needed to test for the generalizability of these findings. Another limitation relates to the year of data collection, which dates back to 2003–2005. Although the considered region in Germany is still characterized by low levels of cultural diversity, immigration has increased in recent years. While a general trend toward more tolerant attitudes toward diversity could be observed over the past decades, political polarization rose at the same time (Follmer et al., 2018). Voices critical of immigration, for example, became distinctively louder in the aftermath of swiftly increased numbers of refugees across Europe in 2015 and, concurrently, right-wing populist parties experienced a significant rise in support (Steinmayr, 2021). To better understand such macrocontextual processes, it would be interesting for future studies to account for the potential workings of societal processes, such as the salience of migration-related issues, and its impact on youth’s attitudes toward immigrants.

## Conclusion

Just as societies become increasingly diverse, strategies aimed at fostering positive intergroup attitudes are needed. This also applies to regions characterized by low levels of cultural diversity and thus limited opportunities for cross-cultural contact. Adolescence is a sensitive period for the development of intergroup attitudes. This development is shaped by experiences in proximal socialization contexts, such as schools. The findings of the present study show that school experiences, such as a democratic classroom climate or supportive relations to classmates, can help reduce the risk of prejudice development. At the same time, they also point at the necessity of accounting for the multilevel nature of the school context and individual student characteristics, such as age. By providing opportunities to learn about social interaction and democratic processes these findings emphasize, once again, that schools matter in youth’s socio-political development.

## Supplementary Information

Online Supplementary Material

## Footnotes

1 Intraclass correlations for negative attitudes toward immigrants at the school level were: ICC_T1_ = 0.10, ICC _T2_ = 0.09, and ICC _T3_ = 0.09. Intraclass correlations for school experiences at T1 were as follows: ICC = 0.05 for perceived multicultural education, ICC = 0.07 for supportive peer relations in class, and ICC = 0.07 for democratic classroom climate.

2 The correlation between age and grade level was *r*_sp_ = 0.92.

3 The following fit indices were considered: Chi-square (*χ*^2^) associated with degrees of freedom (*df*) and *p* value as well as CFI (comparative fit index), TLI (Tucker–Lewis index), RMSEA (root mean square error of approximation), and SRMR (standardized root mean square residual).

4 To account for the MLR estimation of the present analyses, this difference test was conducted using the log-likelihood values and MLR scaling correction factors. The test statistic of this difference test is also chi-square distributed.

5 In follow-up analyses, it was tested whether there were any effects of school experiences on the slope factor at the classroom level which, however, were all far from significance.

6 The covariates age, gender, SES, citizenship status (Level 1) and school track (Level 2) showed only significant effects on the intercept factor. Since there were no significant covariate effects on the slope factor, these effects were constrained to zero in the model estimation.

7 To see whether the changed pattern concerning the effect of perceived multicultural education on the intercept factor at Level 2 might be driven by a particular covariate besides age, follow-up analyses were conducted. Among the Level 1 covariates only gender showed to significantly interact with the Level 2 effect of perceived multicultural education (*B* = 0.785, *SE* = 0.329, *p* = 0.017). However, when accounting for school track at Level 2, the results pointed to a further interplay between school track and gender. More precisely, the results revealed a three-way interaction between gender, school track, and perceived multicultural education at Level 2 (*B* = 0.720, *SE* = 0.295, *p* = 0.015). Accordingly, as shown in Supplementary Table S3, the significant association between perceived multicultural education and negative attitudes toward immigrants at Level 2 was only apparent among male students from higher track schools.
