# Supplementary material for: School Matters: The Effects of School Experiences on Youth’s Attitudes toward Immigrants
Source: J Youth Adolesc. 2021 Sep 24;50(11):2208–23. doi: 10.1007/s10964-021-01497-x (PMC8505319; doi:10.1007/s10964-021-01497-x)
Supplement: Supplementary file 1 — Online Supplementary Material [file 10964_2021_1497_MOESM1_ESM.docx]

**Online Supplemental Material**

Table S1

*Item Wording of Main Study Variables*

| *Negative* A*ttitudes Toward Immigrants*  (adapted from Balke, El-Menouar, & Rastetter, 2002; Dicke, Edinger, & Schmitt, 2000; Kracke & Held, 1994) | |
| --- | --- |
| 1. | Immigrants increase the crime rate. |
| 2. | Immigrants take away the jobs from people who were born in Germany. |
| 3. | Immigrants who work in Germany should eventually go back home. |
| 4. | One always has to be on alert in foreign countries. |
| 5. | Immigrants should choose their spouses among their own nationals only. |
| 6. | Immigrants come to Germany only to exploit our social system. |
|  |  |
| *Perceived Multicultural Education*  (the question was developed within the project) | |
| 1. | Some teachers try very hard to familiarize us with the culture and points of view in other countries. |
|  |  |
| *Supportive Peer Relations in Class*  (Eder, 1998) | |
| 1. | There is a strong sense of community in our class. |
| 2. | If somebody from the class needs help, classmates are eager to help her/ him. |
| 3. | In this class, everyone is only concerned with themselves and their own problems, hardly anyone is interested in the problems of others. |
| 4. | It is a matter of course for us that better students help students who are not so good. |
|  |  |
| *Democratic Classroom Climate*  (adapted from Eder, 1998; Torney-Purta, Lehmann, Oswald, & Schulz, 2001) | |
| 1. | Students feel free to disagree openly with their teachers about political and social issues during class. |
| 2. | Students are encouraged to make up their own minds about issues. |
| 3. | Students feel free to express opinions in class even when their opinions are different from most of the other students. |
| 4. | Teachers respect our opinions and encourage us to express them during class. |
| 5. | Our teachers let us decide many things for ourselves. |
| 6. | Our teachers are fair. |
| 7. | The grades we get are fair. |

Table S2

*Fit Indices of Model Estimations*

| Model | χ²(df) | *p* | CFI | TLI | RMSEA | SRMR_Within_ | SRMR_Between_ |
| --- | --- | --- | --- | --- | --- | --- | --- |
| *Models without Covariates* | | | | | | |  |
| Model 2.1 | 4.29 (6) | .638 | 1.000 | 1.000 | .000 | .005 | .097 |
| Model 2.2 | 3.65 (6) | .723 | 1.000 | 1.000 | .000 | .005 | .087 |
| Model 2.3 | 4.62 (6) | .593 | 1.000 | 1.000 | .000 | .008 | .085 |
| *Models with Covariates* | | | | | | |  |
| Model 3.1 | 9.88 (16) | .873 | 1.000 | 1.000 | .000 | .008 | .099 |
| Model 3.2 | 8.61 (16) | .929 | 1.000 | 1.000 | .000 | .008 | .084 |
| Model 3.3 | 9.34 (16) | .892 | 1.000 | 1.000 | .000 | .009 | .085 |
| *Models with Interaction Terms at Level 2* | | | | |  |  |  |
| Model 5.1 | 9.77 (14) | .778 | 1.000 | 1.000 | .000 | .005 | .092 |
| Model 5.2 | 9.10 (14) | .824 | 1.000 | 1.000 | .000 | .005 | .065 |
| Model 5.3 | 13.44 (14) | .492 | 1.000 | 1.000 | .000 | .008 | .069 |

*Note.* Due to the type = random algorithm, fit indices are not available for Model 4.1 – 4.3. χ² = Chi-square, df = degrees of freedom, *p* = *p*-value, CFI = comparative fit index, TLI = Tucker-Lewis index, RMSEA = root mean square error of approximation, SRMR = standardized root mean square residual. A lower χ² to degrees of freedom ratio (≤ 2.0) or a nonsignificant *p*-value (*p* ≥ .05) indicate a good model fit. While for RMSEA and SRMR values lower than .06 and .08 point to a good fitting model, for CFI and TLI values higher than .95 are desirable (Hu & Bentler, 1999).

Table S3

*Zero-Order Correlations Between Perceived Multicultural Education and Negative Attitudes Toward Immigrants at Time 1 at the Classroom Level by School Track and Gender*

|  |  | Female Students | Male Students |
| --- | --- | --- | --- |
| Lower School Track |  | -.022 | -.129 |
| Higher School Track |  | -.024 | -.673** |

*Note.* * *p* < .05, ** *p* < .01.
